# Advanced Age as a Predictor of Unsuccessful Bilateral Sentinel Lymph Node Detection Using Radiocolloid Mapping in Patients With Endometrial Cancer: A Prospective Observational Study

**DOI:** 10.7759/cureus.84699

**Published:** 2025-05-23

**Authors:** Shinichi Togami, Nozomi Furuzono, Mika Fukuda, Mika Mizuno, Hiroaki Kobayashi

**Affiliations:** 1 Department of Obstetrics and Gynecology, Faculty of Medicine, Kagoshima University, Kagoshima, JPN

**Keywords:** age, bilateral detection, endometrial cancer, radiocolloid mapping, sentinel lymph node

## Abstract

Objective: To identify clinical and pathological factors associated with unsuccessful bilateral sentinel lymph node (SLN) detection using the technetium-99m-labeled phytate radiocolloid method (RI method) in patients with endometrial cancer.

Methods: This prospective observational study included 223 patients with histologically confirmed presumed early-stage endometrial cancer who underwent SLN mapping between July 2018 and May 2022 at Kagoshima University Hospital. A radiocolloid tracer was injected into the cervix the day before surgery, followed by preoperative single-photon emission computed tomography combined with computed tomography imaging. The SLNs were intraoperatively localized using a gamma probe and assessed via frozen sections or one-step nucleic acid amplification. Bilateral SLN detection was defined as successful localization of at least one SLN in each hemipelvis. Multivariate logistic regression was used to identify the independent predictors of detection failure.

Results: The overall bilateral SLN detection rate was 83.4% (186/223). In the univariate analysis, advanced age (median 63.0 vs. 58.0 years, p = 0.0003) and myometrial invasion ≥50% (p = 0.0289) were associated with unsuccessful detection. In the multivariate analysis, age was a significant independent predictor (OR = 1.057, 95% CI: 1.017-1.099, p = 0.0037), whereas other variables, including body mass index, histological type, lymphovascular space invasion, and cervical stromal invasion, were not.

Conclusions: Advanced age significantly increased the risk of unsuccessful bilateral SLN detection using the RI method for endometrial cancer. These findings emphasize the need for individualized surgical planning in older patients, including preoperative counseling and consideration of adjunctive strategies to improve detection rates, particularly in cases in which alternative tracers, such as indocyanine green, are not reimbursed.

## Introduction

Endometrial cancer is one of the most common gynecological malignancies in developed countries, with a steadily increasing incidence in Japan due to aging and lifestyle changes [1]. Accurate staging at the time of diagnosis is essential because lymph node metastasis is a critical prognostic factor that significantly influences treatment decisions and long-term outcomes [2]. Traditionally, systematic lymphadenectomy has been used to evaluate the nodal status; however, this approach is associated with substantial morbidity, including lymphedema, lymphocyst formation, and increased operative time [3].

Sentinel lymph node (SLN) biopsy has emerged as a minimally invasive alternative that enables precise nodal assessment while reducing surgical morbidity. The National Comprehensive Cancer Network guidelines endorse SLN mapping as a viable staging method, particularly in patients with presumed early-stage endometrial cancer [2]. Numerous studies have demonstrated its high sensitivity and negative predictive value using appropriate techniques [4-7].

Although indocyanine green (ICG) fluorescence imaging has been widely adopted in Western countries due to its superior bilateral detection rates [8-10], ICG has not yet been reimbursed under the Japanese national health insurance system for endometrial cancers. Consequently, radiocolloid-based methods using technetium-99m-labeled phytate remain the standard tracer for SLN mapping in Japan [11]. Among these, the use of technetium-99m-labeled technetium phytate (RI method) offers the advantage of preoperative lymphoscintigraphy, single-photon emission computed tomography combined with computed tomography (SPECT/CT), and intraoperative gamma probe detection, facilitating accurate node localization [12,13].

Despite the growing application of SLN biopsy, bilateral detection remains a challenge in a subset of patients, potentially compromising the diagnostic accuracy and requiring conversion to full lymphadenectomy. Identifying the clinical and pathological factors associated with unsuccessful bilateral SLN detection is essential for improving the reliability and applicability of SLN mapping strategies; however, few studies to date have specifically investigated the predictors of unsuccessful bilateral detection. This study aimed to evaluate the risk factors associated with unsuccessful bilateral SLN detection using the RI method with technetium-labeled phytate in patients with endometrial cancer. Understanding these predictors may guide preoperative counseling, surgical planning, and tracer selection, particularly in settings where alternative tracers such as ICG are not yet available.

This work has not been previously presented in any form at conferences or academic meetings.

## Materials and methods

This single-center prospective observational study was conducted at Kagoshima University Hospital to identify the clinical and pathological predictors associated with unsuccessful bilateral SLN detection in patients with endometrial cancer. The study protocol was approved by the Institutional Review Board of Kagoshima University (IRB No. 20-K04), and written informed consent was obtained from all participants. This prospective observational analysis was conducted as part of a larger IRB-approved clinical study investigating SLN-guided management in uterine cancer (IRB title: "Clinical Trial on Omission of Lymphadenectomy Based on Intraoperative Sentinel Lymph Node Diagnosis in Uterine Cancer Surgery").

Study population and eligibility criteria

Between July 2018 and May 2022, consecutive patients with histologically confirmed endometrial cancer who were clinically diagnosed with apparent early-stage disease and scheduled for primary surgical treatment with SLN mapping were prospectively enrolled. All patients enrolled in this study were female. Exclusion criteria were prior pelvic radiation, previous pelvic or para-aortic lymphadenectomy, radiologically suspected distant metastasis, or severe medical comorbidities that precluded surgery.

SLN mapping procedure

SLN mapping was performed using technetium-99m-labeled phytate as a radiotracer. One day before surgery, 1 mCi of the tracer was injected into the cervical stroma in four quadrants (12, 3, 6, and 9 o’clock positions) using a 26-gauge needle to a depth of approximately 1 cm. Following injection, planar lymphoscintigraphy and SPECT/CT were performed to evaluate preoperative SLN localization. Nuclear medicine physicians interpreted the imaging results.

During surgery, a handheld gamma detection probe was used to identify and excise SLNs in the pelvic region. SLNs were intraoperatively evaluated for metastasis using either frozen section histopathology or a one-step nucleic acid amplification (OSNA) assay. Systematic pelvic lymphadenectomy was omitted in patients with negative SLNs and successful bilateral detection. In contrast, patients with SLN metastasis or unsuccessful bilateral SLN detection underwent systematic pelvic lymphadenectomy as determined by the attending gynecologic oncologist.

Data collection and variables

Demographic and clinical data including age, body mass index (BMI), surgical approach, histologic subtype (endometrioid vs. non-endometrioid), depth of myometrial invasion, presence of lymphovascular space invasion (LVSI), and cervical stromal invasion were prospectively recorded. Bilateral SLN detection was defined as the successful identification of at least one SLN in each hemipelvis. All variables were defined according to established clinical and pathological standards. Patients with missing data were excluded from the analysis.

Bias control

Selection bias was minimized by consecutive patient enrollment. Measurement bias was addressed using standardized tracer injection techniques, uniform preoperative imaging protocols, and consistent intraoperative procedures performed by experienced gynecological oncology surgeons.

Sample size justification

This study was exploratory and did not involve formal sample size calculations. All patients who met the inclusion criteria and completed SLN mapping during the study period were included, resulting in 223 evaluable cases.

Statistical analysis

All statistical analyses were conducted using the JMP software (version 14.0; SAS Institute Inc., Cary, NC, USA). Continuous variables were analyzed using the Wilcoxon rank-sum test and reported as medians with interquartile ranges. Categorical variables were compared using the chi-squared test or Fisher exact test, as appropriate. All clinically relevant variables, regardless of their statistical significance in the univariate analysis, were included in a multivariate logistic regression model to identify independent predictors of unsuccessful bilateral SLN detection. Odds ratios (ORs) with 95% confidence intervals (CIs) were calculated, and a p-value < 0.05 was considered statistically significant.

## Results

Bilateral SLN detection was achieved in 186 of 223 patients (83.4%). In the univariate analysis (Table 1), advanced age was significantly associated with unsuccessful bilateral SLN detection (median: 63.0 vs. 58.0 years, p = 0.0003, Wilcoxon rank-sum test). In contrast, BMI, histological type, LVSI, and cervical stromal invasion were not significantly associated. Myometrial invasion ≥1/2 was significantly more frequent in patients with failed bilateral SLN detection compared to those with successful detection (35.1% vs. 51.1%, p = 0.0289). Perioperative outcomes were also analyzed. Three intraoperative complications were observed: one bladder injury, one small bowel injury, and one rectal injury. Postoperative complications were recorded in five patients, including two cases of ileus, two cases of pelvic cavity infection, and one case of ureteral stricture.

**Table 1:** Univariate analysis of predictors of unsuccessful bilateral SLN detection in patients with endometrial cancer (n = 223). BMI, body mass index; LVSI, lymphovascular space invasion; SLN, sentinel lymph node. Statistical analysis was performed using the Mann–Whitney U test for continuous variables and the chi-square or Fisher’s exact test for categorical variables. Statistical significance was set at p < 0.05.

Variable	Bilateral SLN detected (n = 186)	Not bilateral SLN detected (n = 37)	Test statistic	p-value
Age (years)	58 (range: 28–91)	63 (range: 40–81)	Z = 3.652	0.0003
BMI (kg/m²)	25.6 (range: 16.4–50.6)	24.9 (range: 16.7–53.1)	Z = –0.568	0.5702
Histologic type	-	-	χ² = 0.385	0.5348
Endometrioid	176 (94.6%)	34 (91.9%)	-	-
Non endometrioid	10 (5.4%)	3 (8.1%)	-	-
Myometrial invasion	-	-	χ² = 4.776	0.0289
<50%	152 (81.7%)	24 (64.9%)	-	-
≥50%	34 (18.3%)	13 (35.1%)	-	-
LVSI	-	-	χ² = 0.000	0.9895
No	156 (83.9%)	31 (83.8%)	-	-
Yes	30 (16.1%)	6 (16.2%)	-	-
Cervical stromal invasion	-	-	χ² = 0.647	0.4211
No	181 (97.3%)	35 (94.6%)	-	-
Yes	5 (2.7%)	2 (5.4%)	-	-

To identify the independent predictors of unsuccessful bilateral SLN detection, a multivariate logistic regression analysis was performed (Table 2). Age remained a significant independent predictor; each additional year of age increased the odds of unsuccessful bilateral SLN detection by 5.7% (OR = 1.057, 95% CI: 1.017-1.099, P = 0.0037). Although myometrial invasion ≥1/2 showed a trend toward increased risk (OR = 2.545, 95% CI: 1.053-6.826, p = 0.0682), it did not reach statistical significance in the multivariate model. Other variables, such as BMI, histological type, LVSI, and cervical stromal invasion, were not independently associated with bilateral SLN detection failure.

**Table 2:** Multivariate logistic regression analysis for predictors of unsuccessful bilateral SLN detection in patients with endometrial cancer (n = 223). BMI, body mass index; LVSI, lymphovascular space invasion; OR: odds ratio. Statistical analysis was performed using the Mann–Whitney U test for continuous variables and the chi-square or Fisher’s exact test for categorical variables. Statistical significance was set at p < 0.05.

Variable	OR	95% CI for OR (lower–upper)	p-value
Age (years) (per year increase)	1.057	1.017–1.099	0.0037
BMI (kg/m²)	1.000	0.942–1.060	0.9812
Histology (non-endometrioid vs. endometrioid)	0.981	0.228–4.044	0.9575
LVSI (positive vs. negative)	0.644	0.122–1.407	0.1444
Myometrial invasion (≥1/2 vs. <1/2)	2.545	1.053–6.826	0.0682
Cervical stromal invasion (present vs. absent)	1.627	0.268–9.371	0.6196

These findings suggest that advanced age is a robust predictor of bilateral SLN detection failure in patients with endometrial cancer, whereas other pathological characteristics showed no consistent or independent associations.

## Discussion

SLN mapping has emerged as a validated technique to accurately assess lymph node metastases while minimizing surgical morbidity and is recommended in several clinical guidelines [2]. In this prospective study, we investigated the clinical and pathological factors associated with unsuccessful bilateral SLN detection using the technetium-99m-labeled phytate (RI) method in patients with endometrial cancer. Our analysis identified advanced age as an independent predictor of mapping failure, while deep myometrial invasion did not show a significant trend toward increased risk. Other factors, including BMI, histologic subtype, LVSI, and cervical stromal invasion, were not independently associated with bilateral detection failure.

These findings are consistent with previous literature suggesting the influence of patient-related factors on SLN detection rates. Andreika et al. [14] reported that older age, the use of methylene blue alone, and the intraoperative presence of bulky lymph nodes were associated with SLN mapping failure using ICG or blue dye tracers​. Garrett et al. [15] found in a large cohort that higher BMI and non-endometrioid histology, but not age, were independent predictors of failure​. These discrepancies may be attributable to differences in tracer type and detection modalities. Our study, based solely on the RI method, provides a focused evaluation of tracer-specific factors that are less influenced by soft-tissue interference and benefit from SPECT/CT-based localization.

Notably, BMI was not a significant factor in our analysis, a finding that contrasts with studies using near-infrared imaging techniques where obesity often impairs detection [16]. This supports the notion that RI-guided mapping may offer more stable performance across BMI ranges. Similarly, Jankulovska et al. [17] reported a 95.1% intraoperative detection rate using Tc-99m-SENTI-SCINT in a high-BMI population, emphasizing the robustness of the RI method.

Traditional tumor-related prognostic indicators such as histologic type and LVSI did not affect SLN detectability. Although these factors influence recurrence risk and adjuvant therapy decisions, they appear to be less relevant to lymphatic visualization when radiocolloid tracers are used. This distinction highlights the importance of differentiating prognostic factors from those affecting the feasibility of mapping, particularly in the context of tracer-specific surgical workflows.

The clinical implications of our findings are clear: older patients undergoing SLN mapping using the RI method are at a higher risk of failed bilateral detection and may require a tailored surgical approach. This includes preoperative counseling regarding the likelihood of additional lymphadenectomy as well as consideration of intraoperative modifications, such as extended exploration time or adjunctive tracer use, where feasible. In contrast, the RI method demonstrated a stable performance across BMI categories, supporting its continued use in populations with varying body habitus, particularly in settings where ICG is not yet reimbursed.

This study had several limitations. First, as a single-institution study, its external validity of the results may be limited. Second, SLN evaluation was performed using two different intraoperative diagnostic methods (frozen section and OSNA), which may have introduced variability. Third, all procedures were performed by experienced surgeons at a high-volume center, potentially limiting the generalizability of the results to less-specialized institutions. Fourth, although prospectively conducted, this was not a randomized trial, and residual confounding factors cannot be excluded. Finally, while ICG is the most widely used tracer for SLN mapping, technetium-99m may offer a valuable alternative in cases where ICG is unavailable or fails to provide adequate lymphatic visualization. Given the potential utility of technetium-99m in reducing surgical invasiveness and expanding access to SLN biopsy in diverse clinical environments, further validation through multicenter, randomized controlled trials is warranted.

## Conclusions

Despite the application of a standardized RI protocol, bilateral SLN detection remains suboptimal in older patients. This reinforces the importance of considering patient age as a physiological factor that influences lymphatic function, independent of tumor biology or technical expertise. The consistent detection rate observed across the BMI groups supports the continued use of the RI method in diverse clinical populations. Moving forward, optimizing SLN mapping will require not only technical refinement but also improved patient stratification to maximize oncological safety and minimize unnecessary lymphadenectomy.
